# *Arctium lappa* Root Extract Prevents Lead-Induced Liver Injury by Attenuating Oxidative Stress and Inflammation, and Activating Akt/GSK-3β Signaling

**DOI:** 10.3390/antiox8120582

**Published:** 2019-11-24

**Authors:** Ahlam Alhusaini, Laila Fadda, Iman H. Hasan, Hanaa M. Ali, Naglaa F. El Orabi, Amira M. Badr, Enas Zakaria, Abeer M. Alenazi, Ayman M. Mahmoud

**Affiliations:** 1 Pharmacology and Toxicology Department, Faculty of Pharmacy, King Saud University, Riyadh 11451, Saudi Arabia; lfadda@ksu.edu.sa (L.F.); ihasan@ksu.edu.sa (I.H.H.); nelorabi@ksu.edu.sa (N.F.E.O.); amibadr@ksu.edu.sa (A.M.B.); malharbi10@yahoo.com (A.M.A.); 2 Common First Year Deanship, King Saud University, Riyadh 11451, Saudi Arabia; hsameh2312003@yahoo.com; 3 Genetic and Cytology Department, National Research Centre, Giza 12622, Egypt; 4 Department of Pharmacology and Toxicology, Faculty of Pharmacy, Suez Canal University, Ismailia 41522, Egypt; 5 Faculty of Pharmacy, Ain Shams University, Cairo 11566, Egypt; 6 Pharmaceutics Department, College of Pharmacy, King Saud University, Riyadh 11451, Saudi Arabia; dhanahi@ksu.edu.sa; 7 Physiology Division, Zoology Department, Faculty of Science, Beni-Suef University, Beni-Suef 62514, Egypt

**Keywords:** burdock, GSK-3β, lead, DNA damage, oxidative stress

## Abstract

*Arctium lappa* L. (*A. lappa*) is a popular medicinal plant with promising hepatoprotective activity. This study investigated the protective effect of *A. lappa* root extract (ALRE) on lead (Pb) hepatotoxicity, pointing to its ability to modulate oxidative stress, inflammation, and protein kinase B/Akt/glycogen synthase kinase (GSK)-3β signaling. Rats received 50 mg/kg lead acetate (Pb(Ac)_2_) and 200 mg/kg ALRE or vitamin C (Vit. C) for 7 days, and blood and liver samples were collected. Pb(Ac)_2_ provoked hepatotoxicity manifested by elevated serum transaminases and lactate dehydrogenase, and decreased total protein. Histopathological alterations, including distorted lobular hepatic architecture, microsteatotic changes, congestion, and massive necrosis were observed in Pb(II)-induced rats. ALRE ameliorated liver function and prevented all histological alterations. Pb(II) increased hepatic lipid peroxidation (LPO), nitric oxide (NO), caspase-3, and DNA fragmentation, and serum C-reactive protein, tumor necrosis factor-α, and interleukin-1β. Cellular antioxidants, and Akt and GSK-3β phosphorylation levels were decreased in the liver of Pb(II)-induced rats. ALRE ameliorated LPO, NO, caspase-3, DNA fragmentation and inflammatory mediators, and boosted antioxidant defenses in Pb(II)-induced rats. In addition, ALRE activated Akt and inhibited GSK-3β in the liver of Pb(II)-induced rats. In conclusion, ALRE inhibits liver injury in Pb(II)-intoxicated rats by attenuating oxidative injury and inflammation, and activation of Akt/GSK-3β signaling pathway.

## 1. Introduction

Lead (Pb) is a serious environmental pollutant with high emission rate worldwide. It is a non-essential heavy metal widely used in industries, including batteries manufacturing and recycling and other applications such as radiation screening [1,2]. Owing to its high emission rate, Pb contamination has been estimated to cause 540,000 deaths annually [3]. Pb can enter the body through the ingestion of contaminated food or water, absorption through the skin or inhalation; 26 million have been postulated to be at risk of Pb poisoning [4]. Pb has a hazardous health impact and can affect several tissues in human and animal bodies. For instance, liver injury osteoporosis, neurological disorders, and numerous cancers have been linked to the prolonged exposure to Pb [5,6]. Inflammatory mediators and cytokines as well as leukocytosis were positively correlated with the circulating Pb levels in subjects exposed to Pb [7,8]. Besides inflammation, the toxicity of Pb has been mainly linked to its ionic properties and excessive production of reactive oxygen species (ROS). Pb can promote ROS generation, diminish antioxidant defenses, and replace mono- and divalent cations in cellular proteins. Consequently, Pb induces oxidative stress and disrupts cellular enzyme activities, metabolism, ion transport, and signaling pathways [9,10,11]. Therefore, oxidative stress and inflammation underlie the toxic effects of Pb. In this context, Pb-induced hepato- and nephrotoxicity have been associated with oxidative stress [12,13,14].

*Arctium lappa* L., commonly known as burdock, is a widely used medicinal plant. In folk medicine, *A. lappa* is used as a diuretic, antipyretic, antimicrobial, anti-hypertensive, and anti-inflammatory agent. In addition, it has been used in the treatment of hepatitis, gout, and many other inflammatory disorders [15,16,17]. Recent studies have demonstrated the beneficial effects of *A. lappa* polysaccharides in regulating lipid metabolism in diabetic rodents [18] and preventing inflammation in vitro and in vivo [17]. The lignan arctigenin and its glycoside arctiin extracted from *A. lappa* have shown potent anti-inflammatory, anti-viral, and neuroprotective activities [19,20,21,22]. In addition to lignans and polysaccharides, other bioactive constituents of *A. lappa* have attracted attention because of their beneficial medicinal and therapeutic effects [23]. The protective activity of *A. lappa* root extract (ALRE) has been demonstrated against carbon tetrachloride (CCl_4_) and acetaminophen hepatotoxicity in ICR mice [24]. However, its protective effect against Pb hepatotoxicity has not been explored. Therefore, we investigated the potential of ALRE to prevent lead acetate (Pb(Ac)_2_)-induced liver injury, pointing to its ability to modulate oxidative stress, inflammation, and Akt/glycogen synthase kinase (GSK)-3β signaling.

GSK-3 is a serine/threonine kinase downstream of growth factors, insulin, and other major cell signaling pathways. It exists in α and β isoforms and has distinctive functions in different cells [25]. Cell metabolism, proliferation, differentiation, and apoptosis are among the cellular activities regulated by GSK3β [26,27,28]. GSK-3β is active in resting cells and its activity is primarily controlled by Akt/protein kinase B through Ser9 phosphorylation [25]; however, other inactivation methods are also known [29]. While the increased activity of GSK-3β promoted liver injury in rodents [30], its inhibition has been associated with accelerated hepatocyte regeneration in acetaminophen-intoxicated mice [31]. Accordingly, activation of Akt/GSK-3β signaling might play a role in the protective efficacy of ALRE against Pb(II) hepatotoxicity.

## 2. Materials and Methods 

### 2.1. Experimental Animals and Treatments

Twenty-four male Wistar rats (170–180 g) were included in this investigation. The animals were housed in the animal facility under standard conditions (23 ± 2 °C and 50–60% humidity) and were given a free access to a chow diet and water. The experimental protocol and treatments were approved by the Animal Care and Use Committee of the College of Pharmacy, King Saud University (Ethical approval no.: KSU-SE-19-33).

The rats were allocated randomly into four groups (*n* = 6) as follows:

*Group I (Control)*: received intraperitoneal (i.p.) injection of physiological saline and 1% carboxymethyl cellulose (CMC) orally for 7 consecutive days.

*Group II (Pb(II))*: received 50 mg/kg Pb(Ac)_2_ [32] via i.p. injection and 1% CMC orally for 7 consecutive days.

*Group III (Pb(II) + ALRE)*: received 200 mg/kg ALRE [33] dissolved in 1% CMC orally and 50 mg/kg Pb(Ac)_2_ i.p. for 7 consecutive days.

*Group IV (Pb(II) + Vit. C)*: received 200 mg/kg vitamin C (Sigma, St. Louis, MO, USA) [34] dissolved in 1% CMC orally and 50 mg/kg Pb(Ac)_2_ i.p. for 7 consecutive days.

Pb(Ac)_2_ was supplied by Sigma (USA) and dissolved in sterile physiological saline for i.p. injection. ALRE was supplied by GNC Live Well (Warwickshire, UK) and the content of the capsules was suspended in 1% CMC. Vitamin C was obtained from Sigma (St. Louis, MO, USA) and dissolved in 1% CMC for oral administration.

Twenty-four hours after the last treatment (day 8), all animals were sacrificed under anesthesia. Blood samples were collected to prepare serum, and the liver was removed, weighed, and a 10% w/v homogenate was prepared in cold phosphate buffered saline (PBS). Following centrifugation, the clear supernatant was collected for the assay of lipid peroxidation (LPO), nitric oxide (NO), reduced glutathione (GSH), and superoxide dismutase (SOD). Other liver samples were collected on 10% neutral buffered formalin for histological processing, whereas others were kept frozen at −80 °C.

### 2.2. Assay of Liver Function

Serum transaminases (ALT and AST), lactate dehydrogenase (LDH), and total protein levels were determined using Randox (Crumlin, UK) kits according to the provided instructions.

### 2.3. Assay of Oxidative Stress Markers and Antioxidants

LPO was determined in the liver homogenate of all groups as previously described [35] and NO was assayed using Griess reagent [36]. GSH content and SOD activity were determined according to Beutler et al. [37] and Marklund and Marklund [38], respectively.

### 2.4. Assay of C-Reactive Protein (CRP), Pro-Inflammatory Cytokines, and Caspase-3

Serum CRP, TNF-α, and IL-1β levels were determined using R&D (Minneapolis, MN, USA) enzyme-linked immunosorbent assay (ELISA) kits. Caspase-3 was assayed using ELISA kit purchased from Cusabio (Wuhan, China). All assays were performed according to the provided instructions.

### 2.5. Histological Examination

The liver samples collected on 10% neutral buffered formalin were fixed for 24 h. The samples were dehydrated, embedded in paraffin wax, and 5-μm sections were cut. After deparaffinization and rehydration, the sections were stained with hematoxylin and eosin (H&E; Sigma, St. Louis, MO, USA) and examined using a light microscope (Olympus light microscope BX40, Olympus Optical Co., Tokyo, Japan).

### 2.6. Western Blot

Pieces from the liver were homogenized in radioimmunoprecipitation assay (RIPA) buffer containing proteinase and phosphatase inhibitors. The protein concentration in the homogenates was determined using Bradford reagent [39] and 40 µg proteins were subjected to 10% sodium dodecyl sulfate/polyacrylamide gel electrophoresis (SDS/PAGE). The separated proteins were transferred to nitrocellulose membranes which were blocked using 5% skimmed milk in tris buffered saline/tween 20 (TBST). After blocking, the membranes were probed with antibodies against pAkt Ser473, Akt, pGSK-3β Ser9, GSK-3β, and β-actin (Novus Biologicals, Centennial, CO, USA) overnight at 4 °C. The blots were washed three times with TBST and incubated with the secondary antibodies for 1 h at room temperature. The membranes were washed three times with TBST and developed using enhanced chemiluminescence detection kit (BIO-RAD, Hercules, CA, USA). The developed blots were scanned, and the band intensity was quantified using ImageJ (version 1.32j, NIH, USA).

### 2.7. DNA Fragmentation Assay

DNA fragmentation was determined by agarose gel electrophoresis. Quantification of DNA fragmentation was carried out as previously described [40]. Briefly, the tissue samples were lysed and centrifuged to generate fragmented DNA (supernatant) and intact chromatin (pellet). The proteins were precipitated, and the samples were treated with diphenylamine. Absorbance of the developed color was measured at 600 nm and the results were presented as percent of the control.

### 2.8. Statistical Analysis

The results were presented as mean ± SEM (standard error of mean). All statistical comparisons were made by one-way analysis of variance (ANOVA) followed by Tukey’s test and the differences were considered statistically significant at *p* < 0.05. The statistical analysis was carried out using GraphPad Prism 7 (La Jolla, CA, USA).

## 3. Results

### 3.1. ALRE Attenuates Pb(II)-Induced Liver Injury

**Figure 1:**
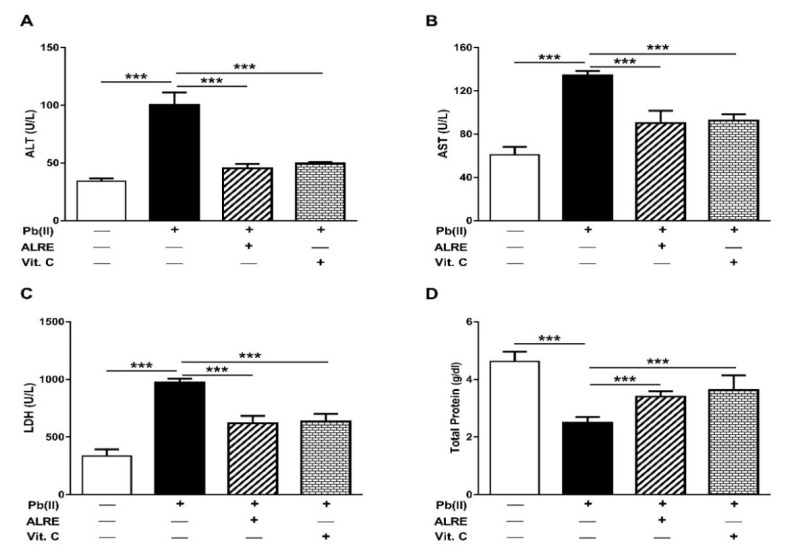
*A. lappa* root extract (ALRE) and Vit. C ameliorate serum ALT (**A**), AST (**B**), LDH (**C**), and total protein (**D**) in Pb(II)-induced rats. Data are expressed as mean ± SEM, (*n* = 6). *** *p* < 0.001.

Pb(II)-intoxicated rats exhibited a significant (*p* < 0.001) elevation in serum ALT, AST, and LDH as depicted in Figure 1A–C. In contrast, serum total protein was significantly declined in Pb(II)-intoxicated rats (*p* < 0.001; Figure 1D). Rats received a concurrent treatment with Vit. C exhibited a significant amelioration of serum transaminases, LDH, and total protein. All the assayed markers were significantly alleviated in Pb(II)-induced rats received ALRE (*p* < 0.001).

**Figure 2:**
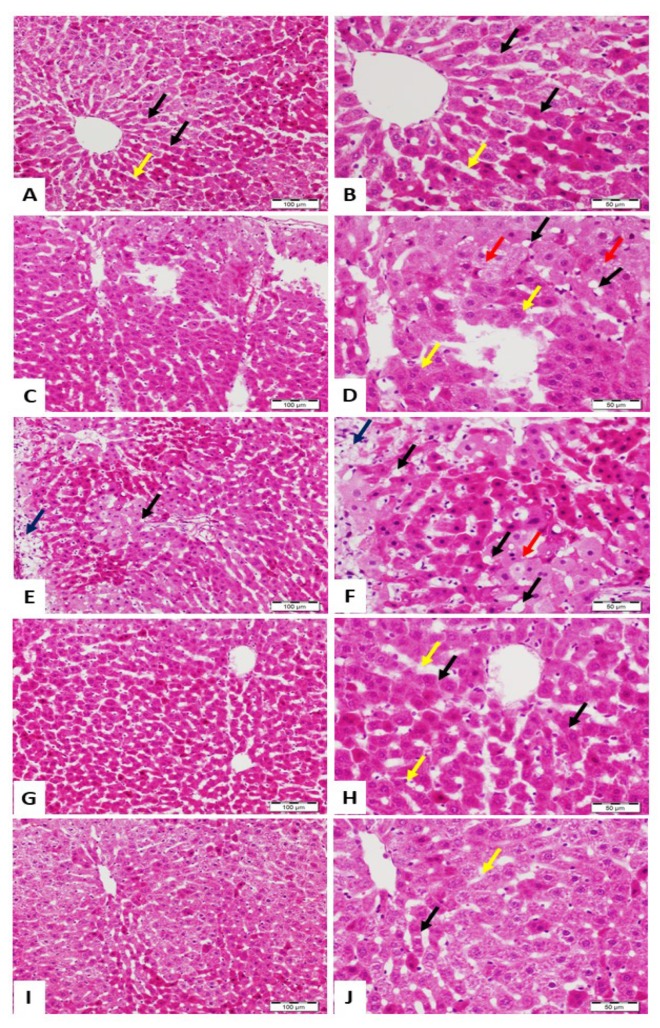
Photomicrographs of hematoxylin and eosin (H&E)-stained sections from liver of (**A**,**B**) control rats showing normal structure and architecture with hepatocytes arranged in thin plates (black arrow), sinusoids (yellow arrow), and central vein; (**C**–**F**) Pb(II)-intoxicated rats showing distorted lobular architecture, ballooning (black arrow), multinucleated hepatocytes (yellow arrow), microsteatotic changes (red arrow), and large areas with necrosis (blue arrow); (**G**,**H**) Pb(II)-administered rats treated with ALRE showing normal hepatic tissue with normal hepatocytes (black arrow) and sinusoids (yellow arrow); and (**I**,**J**) Pb(II)-administered rats treated with Vit. C normal hepatocytes (black arrow) and sinusoids (yellow arrow). (A, C, E, G, and H: ×200, Scale bar 100 µm) and (B, D, F, H, and J: ×400, Scale bar 50 µm).

The ability of ALRE and Vit. C to prevent Pb(II)-induced liver injury was supported by the histological findings (Figure 2). While the control rats showed normal liver structure (Figure 2A,B), Pb(II) provoked multiple histological alterations, including ballooning, distorted lobular hepatic architecture, microsteatotic changes, and massive necrosis (Figure 2C–F). Co-treatment of the rats with ALRE (Figure 2G,H) or Vit. C (Figure 2I,J) prevented all Pb(II)-induced histological changes and the sections showed hepatic tissue with normal architecture and slight congestion of veins at the portal tract.

### 3.2. ALRE Prevents Pb(II)-Induced Oxidative Stress in Liver of Rats

**Figure 3:**
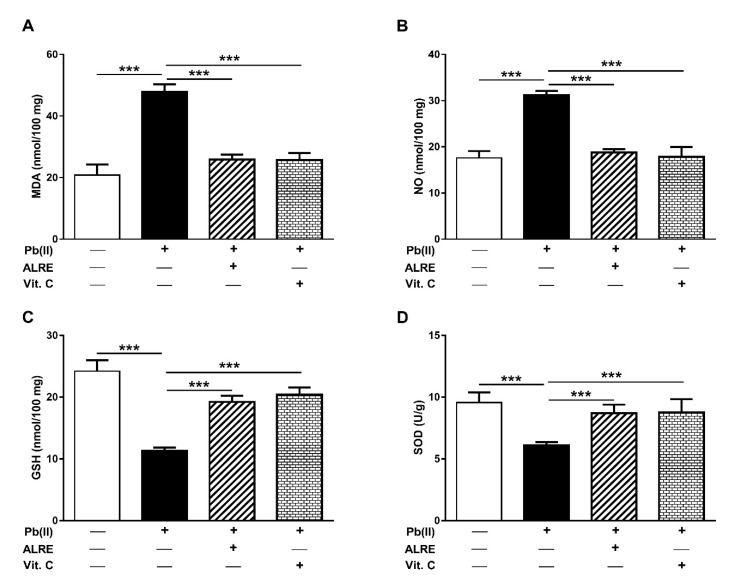
ALRE prevents Pb(II)-induced oxidative stress in the liver of the rats. ALRE and Vit. C decreased malondialdehyde (MDA) (**A**) and nitric oxide (NO) (**B**), and increased glutathione (GSH) (**C**) and superoxide dismutase (SOD) activity (**D**). Data are expressed as mean ± SEM, (*n* = 6). *** *p* < 0.001.

The ameliorative effect of ALRE and Vit. C on Pb(II)-induced oxidative stress was evaluated through the assessment of hepatic LPO, NO, GSH, and SOD. Rats received Pb(II) exhibited a significant increase in hepatic malondialdehyde (MDA; Figure 3A), a LPO marker, as well as NO levels (Figure 3B) when compared with the control group (*p* < 0.001). Concurrent administration of ALRE or Vit. C prevented Pb(II)-induced LPO and NO elevation in the liver of rats.

On the contrary, Pb(II) decreased GSH content (Figure 3C) and SOD activity (Figure 3D) significantly (*p* < 0.001) in the liver of rats when compared with the control group. ALRE and Vit. C boosted both GSH and SOD in the liver of Pb(II)-induced rats.

### 3.3. ALRE Attenuates Inflammation in Pb(II)-Induced Rats

**Figure 4:**
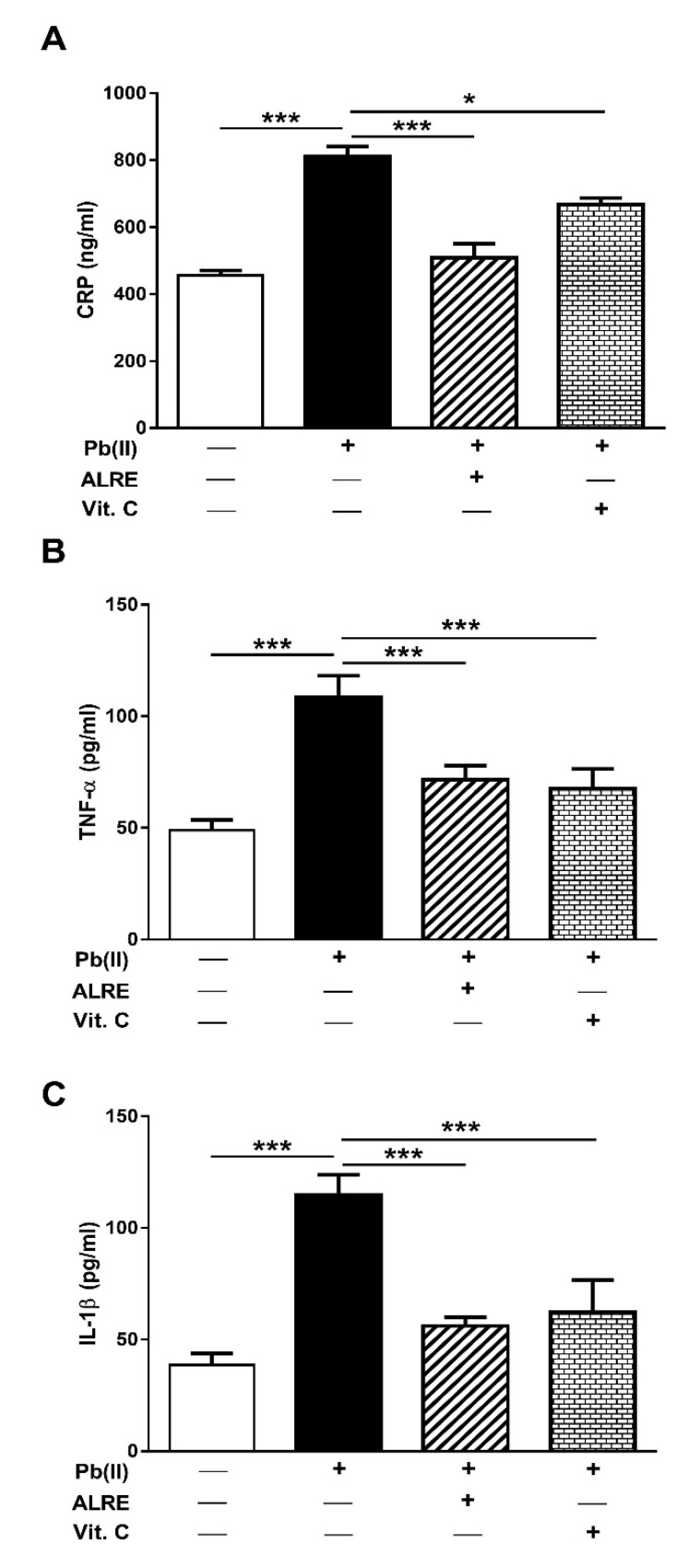
ALRE attenuates inflammation in Pb(II)-induced rats. ALRE and Vit. C decreased serum CRP (**A**), TNF-α (**B**) and IL-1β (**C**) levels in Pb(II)-intoxicated rats. Data are expressed as mean ± SEM, (*n* = 6). * *p* < 0.05 and *** *p* < 0.001.

CRP, TNF-α, and IL-1β were determined in the serum of control and Pb(II)-intoxicated rats. CRP showed a significant elevation in Pb(II)-induced rats when compared with the control group (*p* < 0.001; Figure 4A), an effect that was reversed in rats treated with ALRE (*p* < 0.001) or Vit. C (*p* < 0.05). The circulating levels of the pro-inflammatory cytokines, TNF-α (Figure 4B), and IL-1β (Figure 4C) were markedly increased in Pb(II)-intoxicated rats (*p* < 0.001). Oral supplementation of ALRE or Vit. C to Pb(II)-induced rats decreased serum TNF-α and IL-1β.

### 3.4. ALRE Suppresses Caspase-3 and DNA Fragmentation in Liver of Pb(II)-Induced Rats

**Figure 5:**
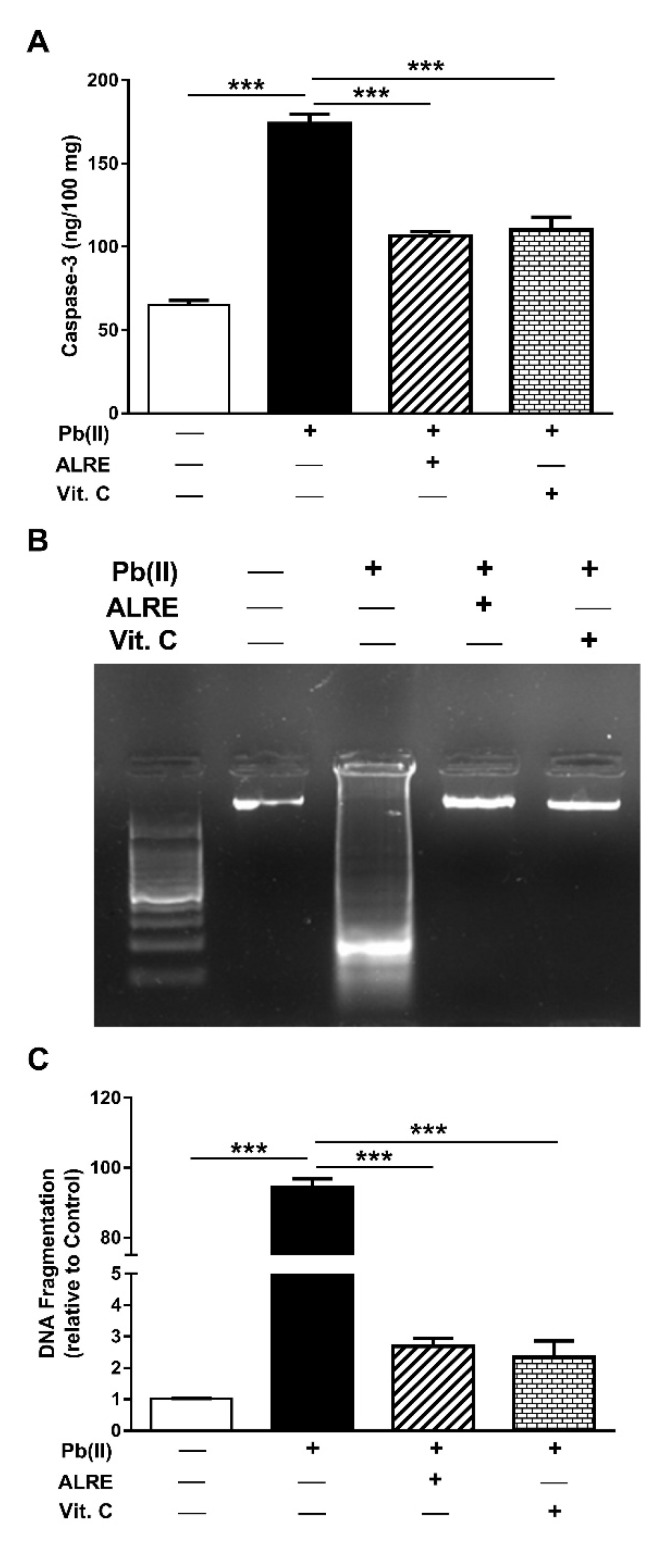
ALRE suppresses caspase-3 and DNA fragmentation in liver of Pb(II)-induced rats. ALRE and Vit. C diminished hepatic caspase-3 (**A**) and inhibited DNA fragmentation (**B**,**C**). Data are expressed as mean ± SEM, (*n* = 6). *** *p* < 0.001.

Caspase-3 was significantly increased in the liver of Pb(II)-intoxicated rats as depicted in Figure 5A. Concurrent administration of ALRE or Vit. C decreased hepatic caspase-3 in Pb(II)-administered rats.

DNA fragmentation was determined by agarose gel electrophoresis and has been spectrophotometrically quantified. The agarose gel electrophoresis revealed noticeable fragmentation of DNA in the liver of Pb(II)-intoxicated rats as represented in Figure 5B. Pb(II)-induced DNA fragmentation was confirmed by the quantitative assay which showed increased DNA fragmentation in the liver of Pb(II)-intoxicated rats (*p* < 0.001). Concomitant administration of ALRE or Vit. C prevented DNA fragmentation in the liver of Pb(II)-induced rats (Figure 5C).

### 3.5. ALRE Activates Akt/GSK-3β Signaling in Liver of Pb(II)-Induced Rats

**Figure 6:**
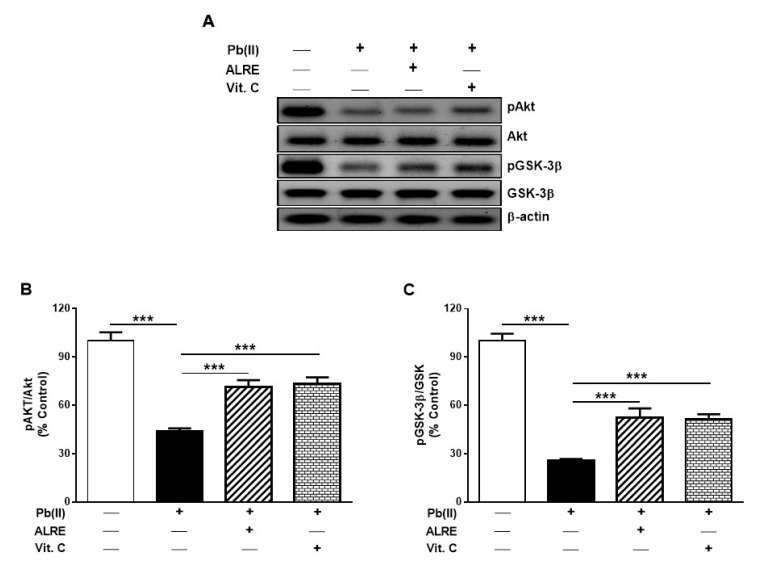
ALRE activates Akt/GSK-3β signaling in liver of Pb(II)-induced rats. (**A**) Representative blots of pAkt, Akt, pGSK-3β, GSK-3β, and β-actin. (**B**,**C**) ALRE and Vit. C increased the levels of pAkt (**B**) and pGSK-3β (**C**). Data are expressed as mean ± SEM, (*n* = 6). *** *p* < 0.001.

Inhibition of GSK-3β has been demonstrated to protect against cell death induced by ischemia/reperfusion (I/R) [41]. The activity of GSK3β is primarily controlled by phosphorylation-mediated inactivation [29]. Therefore, the phosphorylation levels of Akt and GSK-3β were determined to evaluate the involvement of Akt/GSK-3β signaling in the protective effect of ALRE against Pb(II) hepatotoxicity. The data showed significantly reduced pAkt in the liver of Pb(II)-induced rats (Figure 6A,B) when compared with the control animals (*p* < 0.001). Similarly, hepatic pGSK-3β levels were decreased in rats received Pb(II) (Figure 6A,C). Concomitant administration of ALRE or Vit. C increased the phosphorylation levels of Akt and GSK-3β in the liver of Pb(II)-induced rats (*p* < 0.001).

## 4. Discussion

Oxidative stress has been well-acknowledged to be implicated in the toxic effect and tissue injury induced by Pb(II) [12,13,14,42]. *A. lappa* possesses potent antioxidant activity and Lin et al. [24] have demonstrated its ability to reduce MDA, increase GSH and alleviate liver injury in mice challenged with CCl_4_ and acetaminophen. However, nothing is known whether *A. lappa* can modulate Akt/GSK-3β signaling and protect against Pb(II) hepatotoxicity. Here, we showed that ALRE prevents Pb(II)-induced liver injury by attenuating oxidative stress, inflammation and DNA fragmentation, and activating Akt/GSK-3β signaling.

Liver is one of the most common depository sites of Pb within the body [43], and is therefore particularly vulnerable to toxicity and injury. Pb(Ac)_2_ has been well-documented to induce toxicity in different animal models. Accordingly, previous studies have shown increased blood [32] and cerebellar [44] Pb(II) concentration following the i.p. administration of 50 mg/kg Pb(Ac)_2_. Therefore, we used Pb(Ac)_2_-induced rats to study the protective mechanism of ALRE against Pb(II) hepatotoxicity. In this study, administration of 50 mg/kg Pb(Ac)_2_ promoted liver dysfunction and damage manifested by the elevated serum transaminases, LDH, and decreased total protein. The biochemical findings were supported by the histological examination where Pb(II)-intoxicated rats exhibited ballooning, distorted lobular hepatic architecture, microsteatotic changes, central vein congestion, and massive necrosis. In line with these findings, serum transaminases were elevated in rats received 50 mg/kg [32] or 0.4% Pb(Ac)_2_ in drinking water [45] for one and 8 weeks, respectively. ALRE and Vit. C prevented liver dysfunction and histological alterations induced by Pb(II). ALRE has been previously shown to ameliorate serum AST and ALT, and prevented tissue injury induced by CCl_4_ [24] and acetaminophen in mice [24,46]. In cadmium (Cd)-intoxicated rats, ALRE prevented liver injury and ameliorated transaminases [47]. These findings pointed to the potent hepatoprotective efficacy of ALRE.

Given the role of ROS and inflammatory mediators in Pb(II) toxicity, we assumed that attenuation of these pathological processes represents an important part of the hepatoprotective mechanism of ALRE. Our results showed increased MDA and NO accompanied with decreased GSH and SOD in the liver of Pb(II)-intoxicated rats, demonstrating an oxidative stress status. Excess ROS and diminished cellular antioxidants mediate the toxic effects of Pb [9,11]. Pb provokes ROS generations [11], leading to inactivation of enzymatic antioxidants, DNA damage, and cell death [48]. Accordingly, Pb(Ac)_2_ has been reported to elicit cerebellar LPO and reduce SOD activity [44] and alter hepatic antioxidant enzymes gene expression in rats [32]. In addition to oxidative stress, Pb(II) induced inflammation in rats as evidenced by increased serum CRP and the pro-inflammatory cytokines TNF-α and IL-1β. Excess ROS activate the transcription factor nuclear factor-kappaB (NF-κB) that promote the expression of TNF-α, IL-1β, IL-6, and inducible NO synthase (iNOS), and this explains the increased NO levels. The role of inflammation in Pb toxicity has been demonstrated in several studies [7,8,32,49]. In male subjects with high blood Pb and Pb-exposed workers, the inflammatory response has been manifested by leukocytosis and increased inflammatory mediators, including TNF-α [7,8]. In addition, increased TNF-α expression has been reported in blood mononuclear cells treated with Pb(II) and lipopolysaccharide (LPS) [50] and in liver of Pb(Ac)_2_-induced rats [32].

Oral administration of ALRE significantly reduced MDA, NO, and inflammatory mediators, and enhanced the antioxidant defenses in the liver of Pb(II)-induced rats, demonstrating its antioxidant and anti-inflammatory activities. The antioxidant efficacy of *A. lappa* has been demonstrated in previous studies. In CCl_4_- and acetaminophen-induced mice, ALRE decreased hepatic MDA and increased GSH as reported by Lin et al. [24]. ALRE has been suggested to confer its hepatoprotective effects against CCl_4_ and acetaminophen via its antioxidative effect [24]. The same authors have reported the protective effect of *A. lappa* against ethanol/CCl_4_-induced liver injury and attributed the obtained effects to the antioxidant activity of *A. lappa* extract [51]. ALRE has also enhanced antioxidant defenses and prevented liver injury induced by Cd in rats [47]. The antioxidant activity of different extracts of *A. lappa* roots has been demonstrated in vitro where the hydroethanolic extract exhibited the strongest free-radical scavenging efficacy [52]. Besides its antioxidant activity, burdock roots suppressed inflammation both in vivo and in vitro inflammation models [17]. ALRE significantly decreased paw edema induced by carrageenan when administered subcutaneously in rats [53]. In patients with knee osteoarthritis, the consumption of *A. lappa* root tea for 42 days decreased the levels of serum IL-6, CRP, and MDA [54]. The current study added support to the anti-inflammatory efficacy of ALRE. Our findings showed significantly decreased serum CRP, TNF-α, and IL-1β in Pb(II)-induced rats.

The antioxidant and anti-inflammatory activities of *A. lappa* are directly connected to its active phytoconstituents. ALRE has been reported to contain arctigenin, diarctigenin, quercetin, caffeic acid, chlorogenic acid, arctiin, beta-eudesmol, lappaol, polysaccharides, nutrients, and others [17,52]. The antioxidant and anti-inflammatory activities of these active constituents have been demonstrated in several studies. Quercetin, caffeic acid and chlorogenic acid have been well-acknowledged as antioxidant, anti-inflammatory, and hepatoprotective agents [55,56,57]. The water-soluble polysaccharides from *A. lappa* roots increased anti-inflammatory cytokines and diminished TNF-α and IL-1β in macrophages and mice challenged with LPS [17]. Arctigenin inhibited the expression of iNOS, TNF-α, and IL-6 through suppression of NF-κB activation and p65 nuclear translocation in LPS-induced macrophages [22]. Diarctigenin is a lignan that inhibited NO production, suppressed the DNA binding ability of NF-κB, and down-regulated the expression of pro-inflammatory mediators in zymosan-induced macrophages [58]. Arctiin suppressed NF-κB and inhibited the expression of pro-inflammatory mediators in LPS-induced macrophages [59]. In addition to the roots, other parts of *A. lappa* exhibited a potent anti-inflammatory activity. For instance, the hydroethanolic extract of *A. lappa* bark has shown anti-inflammatory activity where it suppressed LPS-induced inflammation and exerted anti-melanoma effects in mice [16]. The methanol extract from the leaves and stem of *A. lappa* suppressed NLRP3 inflammasome and inhibited IL-1β secretion from activated bone marrow derived macrophages [60].

Although the hepatoprotective activity of burdock has been previously reported, the underlying mechanism is not fully understood. We assumed that modulating Akt/GSK-3β signaling might play a role on the hepatoprotective efficacy of *A. lappa*. Herein, we investigated the effect of ALRE on the phosphorylation levels of Akt and GSK-3β in liver of Pb(II)-induced rats. Our results showed that Akt Ser473 and GSK-3β Ser9 phosphorylation levels were significantly decreased in the liver of Pb(II)-intoxicated rats. Interestingly, ALRE supplementation activated Akt/GSK-3β as evidenced by the increased phosphorylation of Akt and GSK-3β. The decreased GSK-3β phosphorylation in liver of Pb(II)-induced rats is a consequence of diminished pAkt. Under resting conditions, GSK-3β is active and its activity is controlled by phosphorylation mediated by Akt as well as other mechanisms [25]. In rodent models of acute liver failure, acetaminophen-induced hepatotoxicity and liver I/R injury, GSK-3β activation has been demonstrated [30,31,41]. Interestingly, inhibition of GSK-3β has been associated with accelerated liver regeneration and inhibition of cell death [30,31,41]. In the present investigation, ALRE activated Akt and suppressed GSK-3β activation, resulting in the inhibition of cell death. The protective effect of ALRE against Pb(II)-induced cell death was further confirmed by inhibition of DNA fragmentation in the liver of rats. The cell death promoting role of GSK-3β is supported by the evidence that activation of PI3K/Akt signaling suppresses apoptosis and inhibits GSK-3β [61]. Furthermore, fibroblasts and neuronal cells apoptosis has been elicited by GSK-3β overexpression and PI3K inhibition, whereas the expression of GSK-3β-K85R, a dominant-negative mutant, prevented cell death [62]. GSK-3 has also been suggested to induce direct phosphorylation and mitochondrial translocation of the pro-apoptotic protein Bax [63]. Therefore, our study provided new information on the role of Akt/GSK-3β signaling in mediating, at least in part, the hepatoprotective effect of ALRE.

In addition to GSK-3β suppression, ALRE exerted a protective effect against Pb(II)-induced cell death by its dual ability to attenuate oxidative stress and inflammation. Excess ROS and activation of inflammatory cascades have been evidenced to induce hepatocyte death [64]. ROS and pro-inflammatory cytokines activate mitochondrial apoptotic pathway resulting in the release of cytochrome *c*, and subsequent activation of caspase-3 and cell death [65]. Accordingly, caspase-3 and DNA fragmentation were increased in the liver of Pb(II)-induced rats, an effect that was inhibited by ALRE supplementation. Hence, it is noteworthy assuming that inhibition of oxidative stress represents a main part of the protective mechanism of ALRE against Pb(II) toxicity and cell death. The ameliorated liver function and attenuated oxidative stress in Pb(II)-intoxicated rats treated with Vit. C supported this assumption. In addition, several studies have reported the protective effects of different antioxidants against drug/chemical-induced hepatocyte apoptosis [64,66,67].

## 5. Conclusions

**Figure 7:**
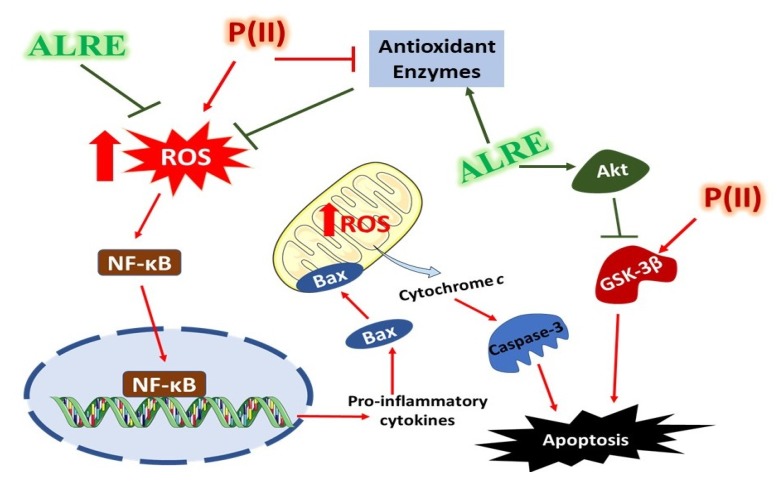
A schematic diagram illustrating the protective mechanism of *A. lappa* root extract (ALRE) on Pb(II)-induced liver injury. Pb(II) provokes reactive oxygen species (ROS) generation which activates NF-κB, and stimulates GSK-3β, resulting in inflammation and cell death via apoptosis. ALRE suppresses ROS production, boosts antioxidant defenses and activates Akt which phosphorylates/deactivates GSK-3β.

This study introduces new information that ALRE prevents Pb(II)-induced liver injury by attenuating oxidative stress, inflammation, and DNA damage and modulating Akt/GSK-3β signaling. Pb(II) promoted oxidative injury, inflammation, and activated GSK-3β, resulting in apoptotic cell death and tissue injury. ALRE attenuated these alterations and activated Akt, resulting in GSK-3β suppression (Summarized mechanistic pathways is presented in Figure 7). The protective effect of ALRE could be attributed to its active constituents; however, further studies scrutinizing the role of each active ingredient and the exact involvement of Akt/GSK-3β signaling are recommended.
